# Ultrasonic bone scalpel for thoracic spinal decompression: case series and technical note

**DOI:** 10.1186/s13018-020-01838-9

**Published:** 2020-08-08

**Authors:** Chuiguo Sun, Guanghui Chen, Tianqi Fan, Weishi Li, Zhaoqing Guo, Qiang Qi, Yan Zeng, Woquan Zhong, Zhongqiang Chen

**Affiliations:** Department of orthopaedics, Peking University Third Hospital, 49 North Garden Road, Haidian District, Beijing, 100191 China

**Keywords:** Complication, High-speed drill, Laminectomy, Ultrasonic bone scalpel, Thoracic spinal stenosis

## Abstract

**Background:**

Thoracic spinal stenosis (TSS) is a rare but intractable disease that fails to respond to conservative treatment. Thoracic spinal decompression, which is traditionally performed using high-speed drills and Kerrison rongeurs, is a time-consuming and technically challenging task. Unfavorable outcomes and high incidence of complications are the major concerns. The development and adaptation of ultrasonic bone scalpel (UBS) have promoted its application in various spinal operations, but its application and standard operating procedure in thoracic decompression have not been fully clarified. Therefore, the purpose of this study is to describe our experience and technique note of using UBS and come up with a standard surgical procedure for thoracic spinal decompression.

**Methods:**

A consecutive of 28 patients with TSS who underwent posterior thoracic spinal decompression surgery with UBS between December 2014 and May 2015 was enrolled in this study. The demographic data, perioperative complications, operation time, estimated blood loss, and pre- and postoperative neurological statuses were recorded and analyzed. Neurological status was evaluated with a modified Japanese Orthopaedic Association (JOA) scale, and the neurological recovery rate was calculated using the Hirabayashi’s Method.

**Results:**

Thoracic spinal decompression surgery was successfully carried out in all cases via a single posterior approach. The average age at surgery was 49.7 ± 8.5 years. The mean operative time of single-segment laminectomy was 3.0 ± 1.4 min, and the blood loss was 108.3 ± 47.3 ml. In circumferential decompression, the average blood loss was 513.8 ± 217.0 ml. Two cases of instrument-related nerve root injury occurred during operation and were cured by conservative treatment. Six patients experienced cerebrospinal fluid (CSF) leakage postoperatively, but no related complications were observed. The mean follow-up period was 39.7 ± 8.9 months, the average JOA score increased from 4.7 before surgery to 10.1 postoperatively, and the average recovery rate was 85.8%.

**Conclusions:**

The UBS is an optimal instrument for thoracic spinal decompression, and its application enables surgeons to decompress the thoracic spinal cord safely and effectively. This standard operating procedure is expected to help achieve favorable outcomes and can be used to treat various pathologies leading to TSS.

## Background

Thoracic spinal stenosis (TSS) is a rare type of stenosis that is exclusively found in East Asians [1]. It is commonly caused by ossification of the posterior longitudinal ligament (OPLL) and ligamentum flavum (OLF) as well as disk herniation (TDH). TSS is an intractable entity compared with the cervical and lumbar stenosis [2]. Surgery is considered the main treatment approach for severe and progressive TSS [3]. En bloc resection of lamina with or without circumferential decompression is a standard surgical technique for the treatment of TSS [4–6]. However, considering that, the caliber of the thoracic spinal canal is relatively narrow and the spinal cord has a tenuous blood supply, this type of surgery is technical challenging and, if not performed properly, may generate poor outcomes [6]. Consequently, precise surgical skills and instruments are required to safely and effectively decompress the spinal cord.

A high-speed rotatory drill and Kerrison rongeurs are frequently used to perform laminectomy and to remove the osseous spinal lesions. However, drilling is a demanding and highly technical process, which may cause spinal cord and nerve injury if handled inappropriately [7]. Complications such as nerve thermal injury, grasping soft tissue, tearing dura mater, and a mechanical injury may occur during drilling [4, 8].

An ultrasonic bone scalpel (UBS) is a device with an oscillating metal tip developed specifically for bone cutting. Using different ultrasonic frequencies, this device can selectively cut hard tissues, leaving the soft-tissue structures such as the dura mater and nerves intact [9]. Technical advantages of this device, such as less tip-bone contact force, self-irrigating cooling system, and no rolling movement, made it a useful instrument for performing procedures near the dura mater and other neural tissue without excessive mechanical and thermal injury [8, 9]. However, to the best of our knowledge, there are no detailed studies to describe how to safely and effectively use UBS for thoracic spinal decompression, and no standard surgical procedure for thoracic decompression with UBS has been established. Since 2014, we have routinely used the ultrasonic osteotomy system developed by SMTP Technology Co., Ltd. Beijing, China, to perform thoracic spinal decompression. Some experience and precautions have been gained in this process. Thus, the aim of this study is to describe our experience and technique note of using UBS and to put forward a standard surgical procedure for thoracic spinal decompression.

## Material and methods

### Patients

A total of 28 patients with TSS who underwent posterior thoracic spinal decompression surgery at the Peking University Third Hospital, China, between December 2014 and May 2015 were enrolled in this study. Informed consent was obtained from all the patients. The study protocol was approved by the Institutional Review Board of the hospital. An ultrasonic bone scalpel was used during surgery. There were 12 men and 16 women, aged between 36 and 65 years (average, 49.7 ± 8.5 years) old; there were 14 cases with OLF, 3 cases with OPLL, 2 cases with TDH, 6 cases with OLF + OPLL, and 3 cases with OLF + TDH. Of the 28 cases, 16 underwent en bloc resection of the lamina and ossified ligamentum flavum, and 12 received circumferential decompression for spinal cord ventral lesions (Table 1).

**Table 1:** Demographics, clinical, and surgical parameters of 28 patients with TSS

No.	Age/gender	Diagnosis (level)	Surgical approach (segments)	BL (ml)	Number of decompressed segments	Time for LA (min)	JOA Scores (11)		Recovery rate (%)	FU (month)	Complications
							Preop	Postop			
1	47/F	OLF (T2-3) + OPLL (T1-5)	LA (T1-5) + CD (T1/2)	750	4	8	4	10	85.7	52	None
2	46/F	OLF (T2-10) + OPLL (T2-7)	LA (T2-10) + CD (T6/7)	800	8	19	5	10.5	91.7	48	CSF leaks
3	55/F	OLF (T4-12) + OPLL (T4-8), skeletal fluorosis	LA (T4-12) + CD (T6/7)	810	8	40	3	9	75.0	47	Left T4 nerve root injury, CSF leaks
4	50/F	OLF (T1-3) + OPLL (C7-T6)	LA (T1-8) + CD (T5/6)	780	7	25	4	9	71.4	34	None
5	60/M	OLF (T9-T11)	LA (T9-12)	320	3	12	5	10.5	91.7	25	None
6	65/M	TDH (T8-9)	LA (T8-9) + CD(T8/9)	260	1	5	6	10	80.0	40	None
7	40/M	OPLL (C7-T4)	LA (C7-T4)	370	5	10	4	10	85.7	32	None
8	57/F	OLF (T9-11) + TDH (T10-11)	LA (T9-11) + CD (T10/11)	410	2	3	3	9.5	81.3	60	None
9	50/F	OPLL (T1-T5)	LA (T1-5) + CD (T1/2)	490	4	14	5	11	100.0	36	None
10	47/F	OPLL (T5-9)	LA (T1-5) + CD (T6/7)	515	4	20	5	10	83.3	40	Left T2 nerve root injury, CSF leaks
11	62/F	OLF (T1-4) + OPLL (C2-T3)	LA (T1-4)	580	3	19	4	10	85.7	27	None
12	44/M	OLF + TDH (T10-11)	LA (T10-11) + CD (T10/11)	280	1	6	4	10	85.7	31	None
13	44/F	OLF (T9-T11)	LA (T9-11)	270	2	5	6	11	100.0	30	None
14	41/M	OLF (T12-L1)	LA (T12-L1)	110	1	3	4	10	85.7	44	None
15	63/F	OLF (T7-10)	LA (T7-10)	280	3	5	5	11	100.0	48	None
16	36/M	OLF (T2-4)	LA (T2-4)	180	2	5	5	10.5	91.7	37	None
17	52/F	OLF (T10-11)	LA (T10-11)	110	1	2	6	11	100.0	50	None
18	46/M	OLF (T8-12)	LA (T8-12)	460	4	5	5	10	83.3	44	None
19	41/M	OLF + OPLL (T5-8)	LA (T5-8) + CD (T6/7)	435	3	10	3	9.5	81.3	49	None
20	58/F	OLF + TDH (T11-L1)	LA (T11-L1) + CD (T11/12)	380	2	7	6	10	80.0	43	None
21	56/F	OLF (T1-8)	LA (T1-8)	350	7	10	4	9	71.4	28	CSF leaks
22	58/F	OLF (T9-T11)	LA (T9-11)	110	2	5	4	10	85.7	37	None
23	50/M	OLF (T9-T11)	LA (T9-11)	130	2	6	5	11	100.0	31	None
24	39/M	TDH (T11-12)	LA (T11-12) + CD (T11/12)	255	1	3	4	10	85.7	46	None
25	41/F	OLF (T9-T11)	LA (T9-11)	210	2	4	6	10.5	90.0	27	None
26	38/M	OLF (T2-4)	LA (T2-4)	110	2	3	5	10	83.3	38	None
27	60/M	OLF (T10-12)	LA (T10-12)	400	2	5	5	9	66.7	48	CSF leaks
28	45/F	OLF (T1-L2)	LA (T1-L2)	2280	13	40	6	10	80.0	39	CSF leaks

Recovery rate (%) = (postoperative JOA score − preoperative JOA score) × 100/(full score (11) − preoperative JOA score)

*M* male, *F* female, *OPLL* ossification of posterior longitudinal ligament, *OLF* ossification of ligamentum flavum, *TDH* thoracic disk herniation, *LA* en bloc resection of lamina and OLF, *CD* circumferential decompression, *BL* blood loss, *CSF* cerebrospinal fluid, *FU* follow-up

### Surgical instruments

**Fig. 1:**
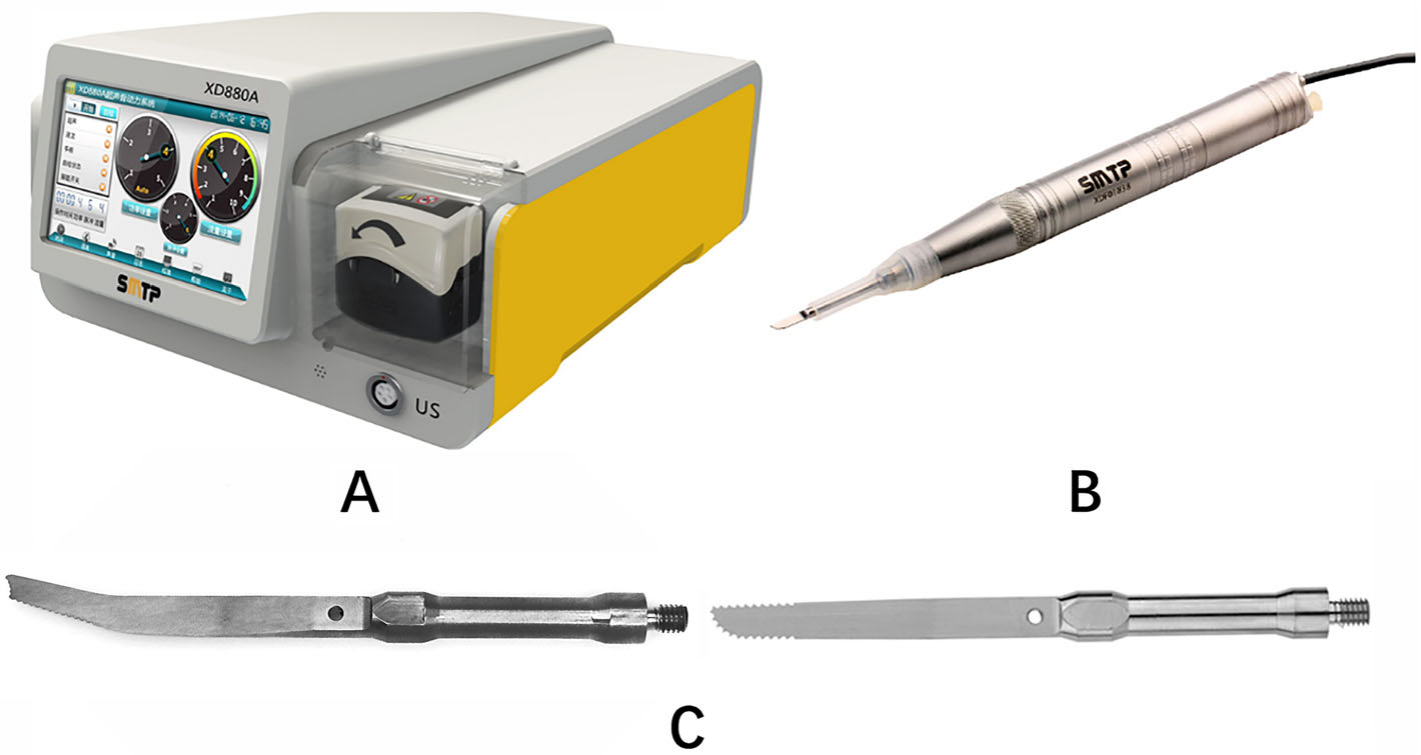
**a** Ultrasonic osteotomy system XD860A. **b** Handpiece equipped with a straight tip. **c** Angled tip (left) and straight tip (right)

Ultrasonic osteotomy system XD860A (SMTP Technology Co., Ltd. Beijing, China) (Fig. 1) was used during surgery. The system included a power supply unit, irrigation pump, footswitch, hand pieces, and tips. The principle of this instrument is to utilize a piezoelectric transducer to convert the electrical input signal into mechanical oscillations so as to achieve efficient bone cutting. The system is integrated with the irrigation pump system, and the irrigation can be delivered near the tips, thus reducing the potential for local thermal necrosis. Two types of tips are commonly used, straight and angled one. The tips move vertically with a range of vibration amplitude of 0–120 μm at a frequency of 39 kHz. The settings for power, frequency modulation, and irrigation rate can be selected on the control panel with a digital display.

### Surgical procedure

**Fig. 2:**
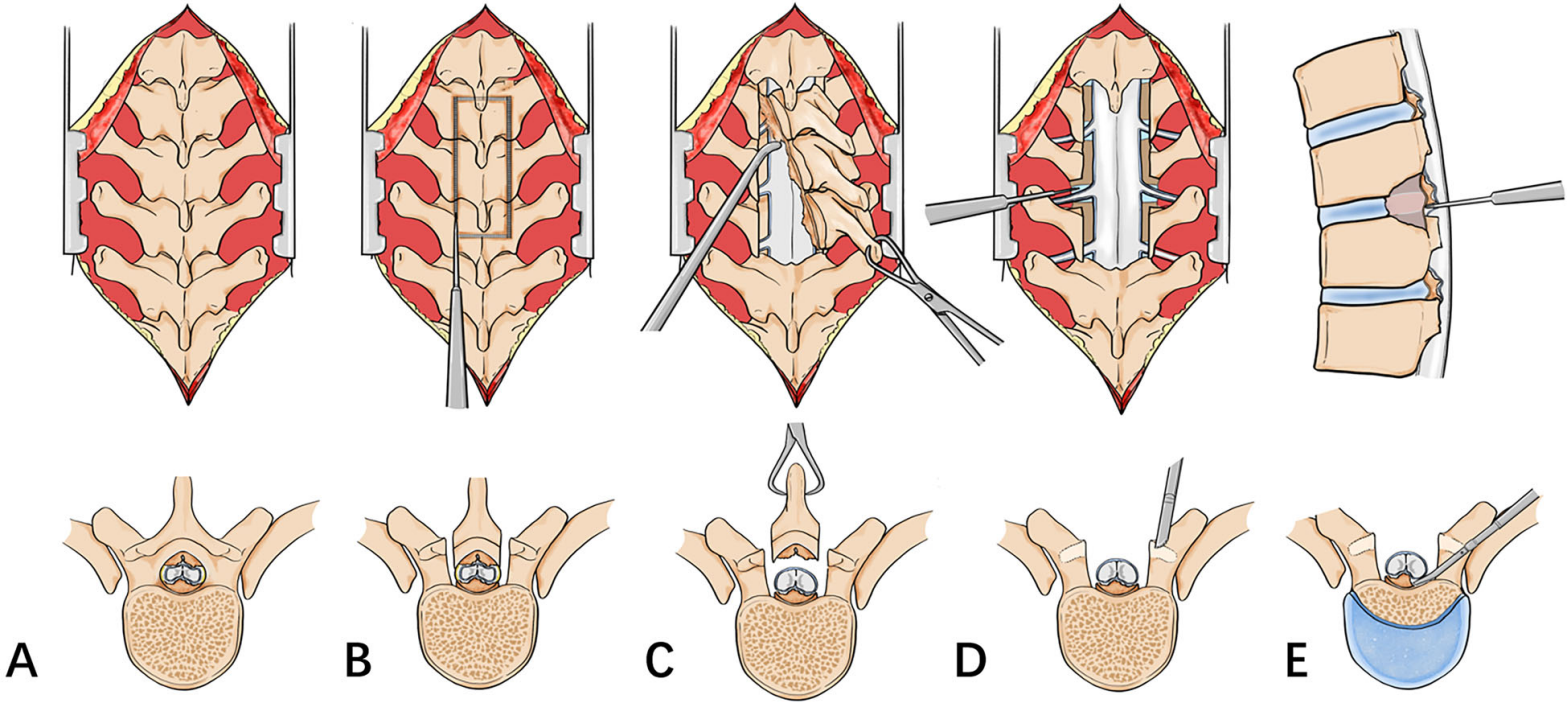
Schematic diagrams of thoracic spinal circumferential decompression with UBS. **a** Expose the lamina and transverse processes of diseased segments. **b** Cut through the lamina and ossified ligament flava along the middle line of bilateral facet joints. **c** Separate the adhesion of ossified ligaments and the dura mater and remove the lamina and OLF integrally. **d** Resect bilateral facet joints and expose the nerve root and intervertebral disk. **e** Remove partial intervertebral disk, create an interconnected passage at the ventral side of OPLL, and thinning, and remove the ossified lesions. UBS, ultrasonic bone scalpel; OLF, ossification of the ligamentum flavum; OPLL, ossification of posterior longitudinal ligament

**Fig. 3:**
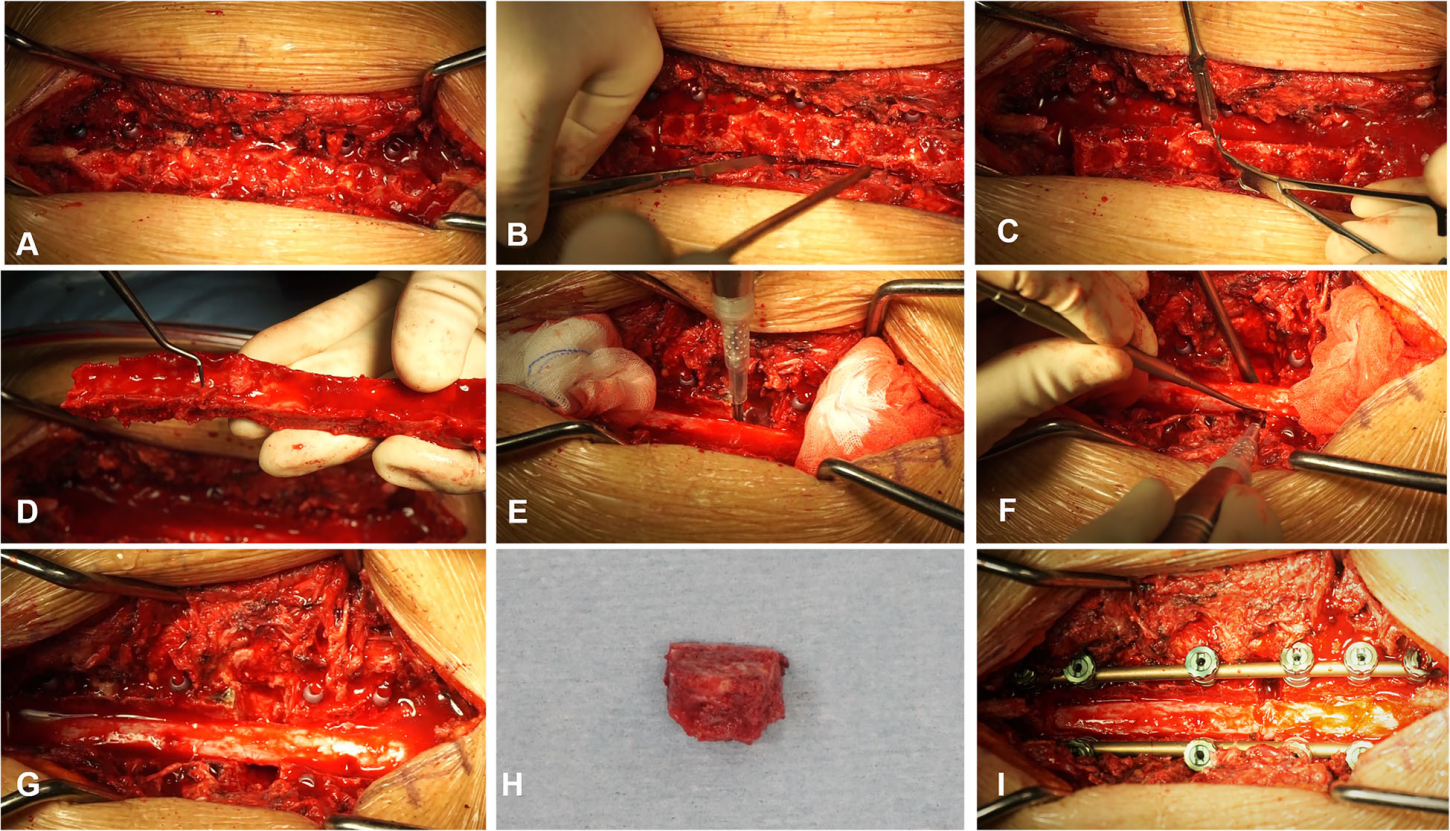
The intraoperative images of a standard operating procedure for circumferential decompression with UBS. **a** Expose the lamina and transverse processes. **b** Pry and amputate the lamina. **c** Separate the adhesion of the ossified ligament and the dura mater. **d** En bloc resection of the lamina and ossified ligamentum flavum. **e** Resect the facet joints. **f** Cut the base of OPLL with a specific designed angled tip. **g** Complete decompression. **h** The resected OPLL mass. **i** Pressurized fixation with screw-rod system

**Fig. 4:**
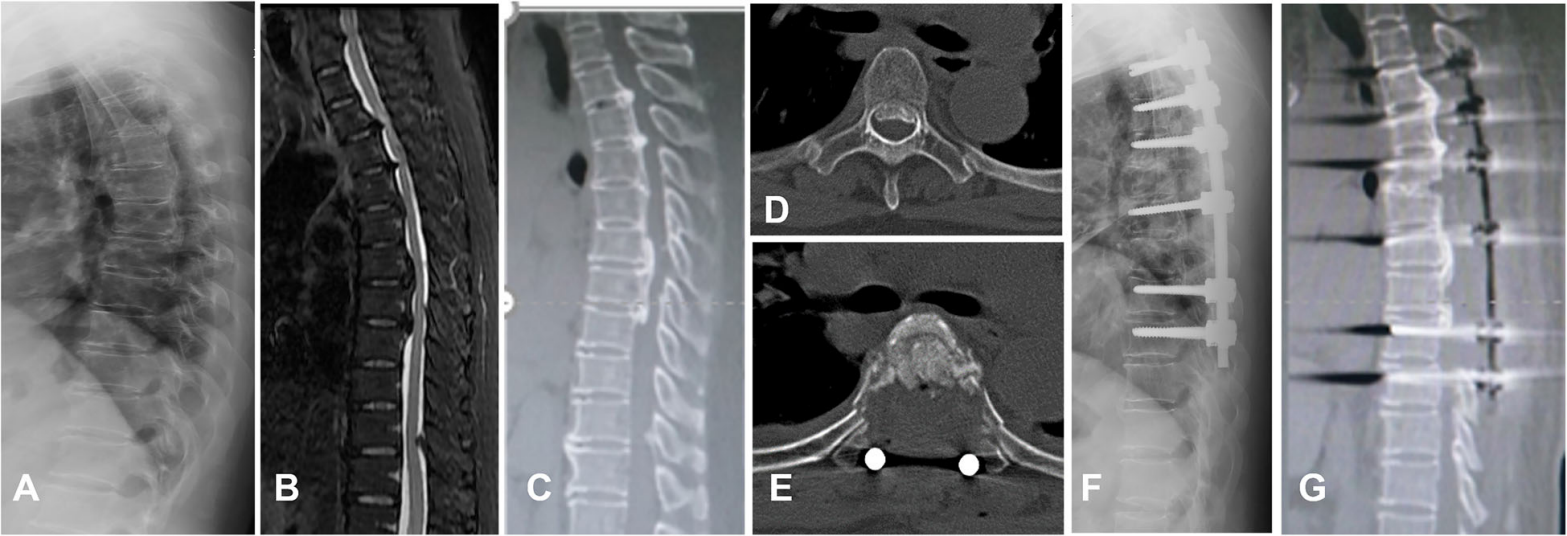
Illustrative case presentation (case 18). **a**–**c** Preoperative X-ray, sagittal MRI, and CT images reveal multiple segment OPLL and isolated OLF in the thoracic spine. **d** Axial CT image indicates decompression segment OPLL. **e** Axial CT image shows complete resection of OPLL. **f**–**g** Postoperative X-ray and CT images show OPLL resection and stable internal fixation. OPLL, ossification of posterior longitudinal ligament; OLF, ossification of the ligamentum flavum

After general anesthesia, patients were placed in a prone position, and a posterior midline incision was performed to expose the lamina and the transverse processes of the diseased segment. The dorsal spinous process, supraspinatus ligament, and interspinous ligament were removed with rongeurs. An ultrasonic bone scalpel equipped with a straight-type tip was used to cut the lamina longitudinally along the midline of bilateral facet joints until the whole layer of bone structure, including the lamina and ossified ligament, was cut through. The cephalad and caudal lamina were then transected. When the lamina and ossified ligaments loosened, a towel forceps was used to catch one end of the lamina and gradually lift it. At the same time, the adhesion of the ossified ligament and the dura mater were separated using a nerve stripper or knife blade. When the lamina is completely lifted, en bloc resection of the lamina and ossified lesions is complete. In cases requiring circumferential decompression, bilateral facet joint excision was performed, which allows space to be created at both sides of the dura sac for subsequent anterior decompression. The posterolateral portion of the intervertebral disk was firstly removed from the bilateral sides of the dura sac, and then an interconnected passage was made at the ventral side of OPLL by gradually removing the residual disk. A specific designed angled tip was used to cut the base of OPLL in an approximately horizontal direction. After exfoliation of adhesions between OPLL and the ventral aspect of the dura sac, the ossified PLL was removed completely (Figs. 2, 3, and 4).

### Follow-ups strategy and evaluation of neurological status

The follow-up strategy was defined from the first day after surgery to the last follow-up. All patients were followed up at 3, 6, and 12 months and then yearly after surgery. The pre- and post-operative neurological status were evaluated according to a modified Japanese Orthopaedic Association (JOA) scale, with a maximum score of 11. The recovery rate was calculated using the Hirabayashi et al. Method [10] (recovery rate (%) = (postoperative JOA score − preoperative JOA score) × 100/(full score (11) − preoperative JOA score)).

## Results

Thoracic spinal decompression surgery was successfully carried out in all cases. A total number of 99 segments underwent laminectomy. The mean operative time for single lamina en bloc resection was 3.0 ± 1.4 min. Intraoperative blood loss was 108.3 ± 47.3 ml in a single-level laminectomy and 513.8 ± 217.0 ml in circumferential decompression.

### Complications

Intraoperatively, two cases of incidental nerve root injury were observed after using an ultrasonic bone scalpel. The incidence of nerve root injury was 7.1% (2/28). In these cases, the nerve root was tightly pinched by ossified ligament and posterior edge of the vertebral body. Therefore, we postulate that the nerve root was injured due to limited cushion space, which occurred due to the contact between the tip and the vertebral body. Moreover, one of the patients with nerve root cutting experienced radiation pain in the left chest wall, which was fully relieved by conservative treatment 4 weeks later.

To overcome this complication, starting with the 11th case in this case series, we adjusted our manipulate procedure for the treatment of severe spinal stenosis by retaining the last thin layer of the ossified lesions instead of penetrating it with UBS and using a narrow osteotome to pry and amputate the lamina. Since then, we have performed more than 300 similar operations without nerve root injury caused by the use of UBS.

Six of the patients have experienced cerebrospinal fluid (CSF) leakage, where five were due to the resection of dura, which was identified ossified intraoperatively, and one case occurred during the separation of OPLL and dura. The dura mater was repaired with a 4-0 silk suture, and muscle or artificial dura were generally used for large dura defects. None of these patients experienced postoperative complications attributable to cerebrospinal fluid leakage.

### Follow-ups and neurological evaluation

The mean follow-up duration was 39.7 ± 8.9 months. Neurological function was improved in all patients. At the last follow-up, the average JOA score increased from 4.7 ± 0.9 before surgery to 10.1 ± 0.6, and the average recovery rate was 85.8%.

## Discussion

Ultrasonic surgical equipment was initially developed in the 1950s when it was mainly applied in dentistry and maxillofacial surgery [11]. The ultrasonic bone scalpel (UBS) is a relatively novel ultrasonic surgical device that cuts bone and spares soft tissues and has been used for various spinal disorders [4, 5, 7, 8]. To date, although the advance of instruments and technique, thoracic spinal decompression surgery is still of great challenging. Unfavorable outcomes and high incidence of complications were frequently reported [12, 13]. To the best of our knowledge, few studies have described how to use UBS for thoracic spinal decompression [4], and no consistent surgical procedures have yet been established. In view of UBS’s technical advantages in bone cutting, we believe that its standard use in decompression surgery will be conducive to achieved success of surgeons. Therefore, the purpose of this study is to present our experience and technique note of using UBS and to put forward a standard surgical procedure for thoracic spinal decompression.

Laminectomy is a well-described technique and has been widely used for treating various spinal stenosis diseases [3]. In some extremely cases with severe spinal cord damage because of the existence of OPLL, it can also be performed as an alternative surgery to circumferential decompression due to its lower risk of postoperative paralysis and technical feasibly [14]. However, in some OLF cases, a standard laminectomy may not completely resect the ossifications, since some of the compression may occur behind the facet joints. In such conditions, an extensive laminectomy including the lamina and the medial half of the bilateral facet joint is required [15]. In this study, we emphasized the decompression width and chose the midline of the articular process as the slotting reference. In addition, choosing this landmark for bone cutting has another technical advantage. Since some authors have suggested that the laminectomy with ultrasonic osteome was performed without direct visualization of dura, and spinal cord injury may occur by insertion of blade beyond the inner cortex of lamina, if penetration of lamina is not felt [4]. In our experience, grooving along the midline of the articular process is safe enough to avoid the possibility of direct contact between the tip and the dura, and even if the blade accidentally penetrates the innermost cortex, the tip will be inserted laterally into the dura without damaging the spinal cord.

En bloc resection of the lamina and ossified ligamentum flavum has many advantages, as it can reduce the operative time and bleeding, and provide a relatively bloodless surgical field. In the past, laminectomy was commonly performed with a high-speed drill and Kerrison rongeurs. The process was technically demanding and time consuming because a large amount of bone was removed piecemeal. Blood loss also increases as the operation time prolonged [3, 5]. Use of UBS for en bloc resection has simplified this surgical procedure and improved the visualization [16]. In this procedure, surgeons only need to cut through the four edges of the lamina and ossified lesions in a relatively safe area, instead of doing operations above the spinal cord like piecemeal resection does, thus significantly reducing the risk of accidental spinal cord injury. By gradually lifting the lamina, the surgeon can quickly assess the adhesion of the ossified lesions to the dura. With the careful operation, iatrogenic durotomy can be avoided in most cases, and the ossified dura can be safely removed under direct vision. Otherwise, intraoperative blood loss is expected to be reduced due to reduced operating procedures and the clotting effect of UBS. Lu et al. [17] compared the efficacy and safety of UBS with high-speed drill for TSS decompression and found that UBS effectively reduced the operation time and blood loss. In this study, the mean blood loss was 108.3 ± 47.3 ml, and the operation time was 3.0 ± 1.4 min for single-segments laminectomy. These results are comparable with previous reports and highlight the feasibility of UBS in saving time and reducing bleeding.

In spite of the effectiveness of laminectomy in most TSS cases, it may not appropriate in all cases, especially those with ventral lesions of the spinal cord such as OPLL and TDH [18]. Circumferential decompression with good surgical outcomes was considered to be an ideal treatment for these diseases. Two-staged posterior-anterior approach and one-stage posterior circumferential decompression have been described [6, 19–21]. However, to the best of our knowledge, there are no consistent surgical procedures have been established due to the rarity of the disease and variety of its complications. The use of UBS in circumferential decompression was less described and is expected to reduce most complications. In this study, UBS was used for one-stage circumferential decompression, bilateral facet joints were excised accurately, and nerve roots were preserved. By creating a buffer space beneath the ventral lesion, we can safely cut the OPLL base using our specially designed angled tip. After separating the adhesion between the dura and OPLL, OPLL was completely removed. During this process, we encountered no nerve injury complications except for an unintended dura tear during the separation of dura adhesions in one patient. The incidence of complications was noted to be lower in comparison with studies with conventional instruments [12, 22, 23].

Dural tear and neurologic deficit are the most common complications of spinal surgery [22, 23]. It is reported that the incidence of durotomy varies greatly ranging from 1.1 to 17% [24], and the rate of neurologic deficit ranges from 8.4 to 19.8% [23]. Risk factors analysis showed that the most common instrument leading to durotomies was the Kerrions punch followed by the curate and then the drill [24]. Liu et al. [5] compared the safety and efficacy of UBS and high-speed drill in multilevel thoracic OLF; the incidence of durotomies was 5.6% and 13.0% respectively. Chen et al. [25] reported 2 cases (12.5%) of dural tear in the high-speed burr group, while no dural injury occurred in the UBS group. In this cohort, six patients have experienced CSF leakage, of which 5 cases were due to the resection of ossified dura mater and the other case is occurred during the separation process of OPLL and dura mater. Although the dura mater of these 5 cases was resected purposely, the fact that patients with dural ossification has a higher incidence of CSF leakage reminding us the importance of preoperative diagnosis and intraoperative management of such cases. In addition, we encountered two cases instrument-relate nerve root injury, with an incidence of 7.1%. The incidence was comparable to previous reports [12, 22, 23]. One explanation is that these complications are mainly occurred at the beginning use of UBS, when surgeons had limited experience with the instrument. Another reason is that both patients were diagnosed with severe TSS, one with OLF + OPLL, also with skeletal fluorosis, and the other with OPLL. In these two cases, the nerve roots were tightly pinched by ossified lesions and vertebral body. During laminectomy, the blade penetrates the inner cortex of the ossified lesion, and the vibratory force was transmitted directly to the dura mater of the nerve root, resulting in root injury. To overcome this complication, we modified our surgical procedure by retaining the last thin layer of ossification, rather than penetrating it, and use a narrow bone osteotome to pry open and amputate the lamina. Since then, we have used this method to perform more than 300 cases of similar surgery, and no UBS related nerve root injuries occurred. This surgical procedure was proved to be safe enough.

In terms of the neurological recovery, all patients have experienced an uneventful recovery process, and the average JOA score was improved from 4.7 ± 0.9 before surgery to 10.1 ± 0.6 at the last follow-up. The average recovery rate was 85.8%, which is much higher than previous reports [3, 6, 12, 26, 27]. The results indicated that with this surgical procedure, postoperative long-term neurological recovery could be achieved.

Overall, for the first time, we systematically described the surgical procedures for the treatment of TSS with UBS. The results of this study were comparable with previous reports in which the authors compared safety and efficiency of using UBS and conventional instruments. In addition, two cases of nerve root injury occurred at the user’s beginning use of UBS, reminding us of the learning curve is required to safely use this novel device. Thus, we believed that successful decompression surgery requires not only appropriate tools but also experience. Before surgeons becoming comfortable with the use of UBS, a system training process is essential.

## Limitations

There are limitations in this study. Firstly, we present a retrospective study, and there is no control group. Secondly, small number of patients makes it difficult to draw statistically significant conclusions. Thirdly, various pathologies leading to TSS were involved in this study, and the indications of surgical procedures for different conditions were not discussed. A further detailed surgical protocols and indications for each pathology should be separately discussed in the future studies. Despite the limitations, favorable outcomes have been achieved with the use of UBS, and this surgical procedure is proved to be reliable for treating TSS. To fully elucidate the potential advantages and complications of using UBS for thoracic spinal decompression, a large-scale prospective cohort study and comparative study with the traditional instruments are required.

## Conclusion

The UBS is an ideal instrument for thoracic spinal decompression, and its application enables surgeons to decompress the thoracic spinal cord safely and effectively. This standard operating procedure is expected to help achieve favorable outcomes and can be used to treat various pathologies leading to thoracic spinal stenosis. To further explore the potential advantages and disadvantages of using UBS and the effectiveness of this surgical procure, a large-scale prospective comparative study is needed.

## Abbreviations

TSS: Thoracic spinal stenosis
UBS: Ultrasonic bone scalpel
OPLL: Ossification of the posterior longitudinal ligament
OLF: Ossification of ligamentum flavum
TDH: Thoracic disk herniation

## References

[1] Gao R, Wen Y, Yang L (2013). Clinical features and surgical outcomes of patients with thoracic myelopathy caused by multilevel ossification of the ligamentum flavum. Spine J.

[2] Hou X, Sun C, Liu X (2016). Clinical features of thoracic spinal stenosis-associated myelopathy: a retrospective analysis of 427 cases. Clin Spine Surg.

[3] Jia LS, X-s C, S-y Z (2010). En Bloc Resection of lamina and ossified ligamentum flavum in the treatment of thoracic Ossification of the ligamentum flavum. Neurosurgery..

[4] Kim CH, Renaldo N, Chung CK (2015). Use of an ultrasonic osteotome for direct removal of beak-type ossification of posterior longitudinal ligament in the thoracic spine. J Korean Neurosurg Soc.

[5] Liu XW, Li TF, Shi L (2019). Application of piezosurgery in en bloc laminectomy for the treatment of multilevel thoracic ossification of ligamentum flavum. World Neurosurg.

[6] Kato S, Murakami H, Demura S (2012). Novel surgical technique for ossification of posterior longitudinal ligament in the thoracic spine. J Neurosurg Spine.

[7] Kim CH, Chung CK, Choi Y, et al. The efficacy of ultrasonic bone scalpel for unilateral cervical open-door laminoplasty: a randomized controlled trial. neurosurgery. 2019; undefined:undefined. 10.1093/neuros/nyz301 31435653

[8] Nakagawa H, Kim S-D, Mizuno J (2005). Technical advantages of an ultrasonic bone curette in spinal surgery. J Neurosurg Spine.

[9] Paolo C, Cavallo LM, Isabella E (2010). Bone removal with a new ultrasonic bone curette during endoscopic endonasal approach to the sellar-suprasellar area: technical note. Neurosurgery.

[10] Hirabayashi K, Miyakawa J, Satomi K (1981). Operative results and postoperative progression of ossification among patients with ossification of cervical posterior longitudinal ligament. Spine.

[11] Sawamura Y, Fukushima T, Terasaka S (1999). Development of a handpiece and probes for a microsurgical ultrasonic aspirator: instrumentation and application. Neurosurgery..

[12] Onishi E, Yasuda T, Yamamoto H, Iwaki K, Ota S (2016). Outcomes of surgical treatment for thoracic myelopathy: a single-institutional study of 73 patients. Spine (Phila Pa 1976).

[13] Shiro I, Kei A, Kazuhiro T (2018). Perioperative complications after surgery for thoracic ossification of posterior longitudinal ligament: a nationwide multicenter prospective study. Spine..

[14] Masashi Y, Makondo M, Yoshikazu I (2006). Clinical results of surgery for thoracic myelopathy caused by ossification of the posterior longitudinal ligament: operative indication of posterior decompression with instrumented fusion. Spine..

[15] Xin-Zhi S, Zhong-Qiang C, Qiang Q (2011). Diagnosis and treatment of ossification of the ligamentum flavum associated with dural ossification: clinical article. J Neurosurg Spine.

[16] Parker Scott L, Kretzer Ryan M, Recinos Pablo F (2013). Ultrasonic BoneScalpel for osteoplastic laminoplasty in the resection of intradural spinal pathology: case series and technical note. Neurosurgery.

[17] Lu X-D, Yi-Bo Z, Xiao-Feng Z (2019). Efficacy and safety analysis of ultrasonic bone curette in the treatment of thoracic spinal stenosis. Orthop Surg.

[18] Satoshi K, Hideki M, Satoru D (2020). Indication for anterior spinal cord decompression via a posterolateral approach for the treatment of ossification of the posterior longitudinal ligament in the thoracic spine: a prospective cohort study. Eur Spine J.

[19] Norio K, Katsuro T, Hideki M (2008). Circumspinal decompression with dekyphosis stabilization for thoracic myelopathy due to ossification of the posterior longitudinal ligament. Spine..

[20] Tomita K, Kawahara N, Baba H, Kikuchi Y, Nishimura H (1990). Circumspinal decompression for thoracic myelopathy due to combined ossification of the posterior longitudinal ligament and ligamentum flavum. Spine..

[21] Tsuzuki N, Hirabayashi S, Abe R, Saiki K (2001). Staged spinal cord decompression through posterior approach for thoracic myelopathy caused by ossification of posterior longitudinal ligament. Spine..

[22] Osman Nebiyu S, Cheung Zoe B, Hussain Awais K (2018). Outcomes and complications following laminectomy alone for thoracic myelopathy due to ossified ligamentum flavum: a systematic review and meta-analysis. Spine.

[23] Xu N, Yu M, Xiaoguang L (2017). A systematic review of complications in thoracic spine surgery for ossification of the posterior longitudinal ligament. Eur Spine J.

[24] Mcmahon P, Dididze M, Levi AD (2012). Incidental durotomy after spinal surgery: a prospective study in an academic institution - Presented at the 2012 Joint Spine Section Meeting. Clinical article. J Neurosurg Spine.

[25] Chen Y, Chang Z, Yu X, et al. Use of ultrasonic device in cervical and thoracic laminectomy: a retrospective comparative study and technical note. Sci Rep. 2018;8(1). 10.1038/s41598-018-22454-y PMC5838170 29507350

[26] KatoSatoshi MH, Satoru D (2015). Gradual spinal cord decompression through migration of floated plaques after anterior decompression via a posterolateral approach for OPLL in the thoracic spine. J Neurosurg Spine.

[27] Yu S, Wu D, Fengning L (2013). Surgical results and prognostic factors for thoracic myelopathy caused by ossification of ligamentum flavum: posterior surgery by laminectomy. Acta Neurochir.

